# Peripheral blood indices to predict PFS/OS with anlotinib as a subsequent treatment in advanced small-cell lung cancer

**DOI:** 10.20892/j.issn.2095-3941.2020.0727

**Published:** 2021-07-24

**Authors:** Cuicui Zhang, Jing Wang, Xinyue Wang, Zhaoting Meng, Ying Cheng, Kai Li

**Affiliations:** 1 Department of Thoracic Oncology Tianjin Medical University Cancer Institute and Hospital, National Clinical Research Center for Cancer, Key Laboratory of Cancer Prevention and Therapy, Tianjin, Tianjin’s Clinical Research Center for Cancer, Tianjin 300060, China; 2 Jilin Cancer Hospital, Changchun 130021, China

**Keywords:** Small-cell lung cancer, anlotinib, predictive factors, PFS, OS

## Abstract

**Objective:**

In the phase II ALTER-1202 (NCT03059797) trial, anlotinib significantly improved progression-free survival (PFS) and overall survival (OS) in patients with advanced small-cell lung cancer (SCLC) who underwent at least 2 previous chemotherapy cycles, when compared with a placebo group. To identify potential factors for predicting efficacy and prognosis with anlotinib treatment, we analyzed hematological indices at baseline and adverse events (AEs) over the course of anlotinib treatment.

**Methods:**

Data were collected from March 2017 to April 2019 from a randomized, double-blind, placebo-controlled, multicenter, phase II trial of anlotinib. Eligible patients were randomly assigned 2:1 to receive anlotinib or placebo until disease progression, intolerable toxicity, or withdrawal of consent. The patients received anlotinib (12 mg) or an analogue capsule (placebo) orally once daily for 14 days every 3 weeks. The hematological indices at baseline and AEs that occurred in the initial 2 treatment cycles were recorded. The Kaplan-Meier test and Cox regression model were used to assess survival differences.

**Results:**

A total of 82 patients (81 patients with complete data) were randomly assigned to receive anlotinib, with 38 receiving a placebo as a control. Multivariate analysis indicated that an elevated neutrophil to lymphocyte ratio > 7.75 and lactate dehydrogenase > 254.65 U/L at baseline were independent risk factors for PFS; basal elevated aspartate aminotransferase > 26.75 U/L, neuron specific enolase > 18.64 ng/mL, and fibrinogen > 4.645 g/L were independent risk factors for OS. During treatment, elevated γ glutamyltransferase and hypophosphatemia were independent predictors for a poor PFS, and elevated γ-glutamyl transferase and hypercholesterolemia were independent factors for OS.

**Conclusions:**

Our study preliminarily defined potential factors that affected the PFS and OS at baseline and during anlotinib treatment in patients with advanced SCLC. Our findings provide a basis for screening the dominant population and for dynamic efficacy monitoring with anlotinib therapy.

## Introduction

Lung cancer is the leading cause of cancer mortality worldwide. Small-cell lung cancer (SCLC) is a highly aggressive neuroendocrine neoplasm that accounts for approximately 13% of all lung cancers and is characterized by rapid disease progression and early metastasis^1^. Cisplatin/carboplatin and etoposide have been the canonical first-line treatments for more than 30 years. Although 35%–86% of patients respond well to first-line chemotherapy, resistance to treatment emerges rapidly and second-line treatments show poor efficacy^2^. However, there is no standard recommendation for third-line treatment, and the benefits of further lines of therapy remain unknown. Anlotinib (AL3818) hydrochloride is a novel multi-targeted tyrosine kinase inhibitor that targets vascular endothelial growth factor (VEGF) receptors (VEGFR1, VEGFR2/KDR, VEGFR3), c-Kit, platelet derived growth factor-α, and fibroblast growth factor receptors (FGFR1, FGFR2, and FGFR3)^3^. Furthermore, anlotinib has been shown to inhibit tumor growth^4^. As a third-line or subsequent therapy, anlotinib was well tolerated and provided improved progression-free survival (PFS) and overall survival (OS) among Chinese patients with non-small cell lung cancer (NSCLC)^5^. In addition, as a third-line or subsequent treatment for SCLC, anlotinib showed a longer PFS (> 4 months) and OS (> 7 months) than the placebo with a favorable safety profile^6^, so it became the first drug approved for third- or further-line treatment in SCLC patients in China (approved on August 30, 2019). In the present study, we reviewed patients treated with anlotinib from a phase II trial to evaluate the main factors [including hematological index at baseline and adverse events (AEs) during treatment] affecting PFS and OS, with the goal of identifying a marker for predicting anlotinib efficacy in patients with SCLC.

## Materials and methods

### Eligibility and exclusion criteria^6^

This trial was performed at 11 sites in China from March 2017 to April 2019. The major inclusion criteria were: (1) patients who were pathologically diagnosed with advanced SCLC (stages IIIa, IIIb, and IV, including refractory cases) and had measurable nidus; (2) patients aged 18–75 years with an Eastern Cooperative Oncology Group performance status of 0–2; (3) patients with disease progression after at least 2 lines of chemotherapy; (4) patients with adequate major organ function within 7 days before enrollment, including an absolute neutrophil count ≥ 1.5 × 10^9^/L and a platelet count ≥ 80 × 10^9^/L, with adequate kidney and liver function, etc.; and (5) patients with normal cardiac function assessed by an echocardiogram as a left ventricular ejection fraction ≥ 50%. The exclusion criteria included: (1) previous treatment with anlotinib or other vascular-targeted therapies, (2) uncontrolled hypertension (systolic blood pressure ≥ 150 mmHg, diastolic pressure ≥ 100 mmHg), (3) active bleeding from any site, and (4) other severe illnesses. The protocol was approved by the ethics committees (Approval No. E2017093) at every site and complied with Good Clinical Practice guidelines and the Declaration of Helsinki. All patients provided written informed consent before enrollment.

### Therapy schedule and follow-up

Patients were randomized at a 2:1 ratio into anlotinib or placebo analogue capsule treatment groups. Because anlotinib has been approved for clinical use in many cancers including NSCLC, we chose the “2:1 ratio” for randomization in parallel with many other trials^7^. The medication was administered orally once daily from days 1–14 in a 21 day cycle, with an initial dose of 12 mg. The dose was reduced to 10 mg or 8 mg in cases of toxicity, according to the protocol. Treatment was continued until disease progression, according to Response Evaluation Criteria in Solid Tumors, version 1.1, occurrence of intolerable toxicity, or withdrawal of consent. Patients were followed-up as per the trial protocol and data were recorded accordingly. Patients were followed-up until disease progression, and were then followed-up every 4 weeks until death or until the study end date.

### Data collection

No more than 1 week prior to enrollment, the baseline hematological index was collected, including routine blood examination, biochemical data, blood coagulation indices, etc. We adopted the commonly used method of using a receiver operating characteristic (ROC) curve to calculate cut-off values for survival^8–12^. PFS and OS measurements were obtained using survival status (dead or alive), and imaging findings were used to determine condition (progression or stable) at the end of the follow-up; these measurements were considered as outcome variables and were also compared to their respective normal clinical values to confirm reliability. The patients were divided into 2 groups according to the cut-off values. AEs were defined as any adverse event, unintended symptom, or abnormal laboratory finding that occurred during treatment with anlotinib, even if drug dosage did not necessarily have a causal relationship with the AE. All AEs that occurred within 2 cycles were assessed according to the guidelines of the Common Terminology Criteria for Adverse Events (CTCAE), version 4.03. Elevated γ glutamyltransferase (GGT) was defined when GGT after anlotinib treatment was higher than the upper limit of normal for each laboratory or baseline value. Because these were enumeration data, patients were categorized into 2 groups: patients with AE and patients without AE. We then compared differences in the PFS and OS between the 2 groups using the Kaplan-Meier method.

### Blood collection

Blood samples were collected at trial sites at baseline and then again on the 7th, 15th, 21st, 42nd, and 63rd day of anlotinib or placebo treatment, with the patient’s consent. This trial was registered with ClinicalTrials.gov, number NCT03059797. All blood samples were anticoagulated with EDTA, stored at 4 °C before use, and tested within 6 h after collection. Other routine indices were tested by laboratory technicians at each hospital. The percentages of CD3^+^T, CD4^+^T, CD8^+^T cells, and natural killer cells in the peripheral blood were analyzed by flow cytometry at Tianjin Medical University Cancer Institute and Hospital. Data from each sample were analyzed by Software-System II (version number EPICS-XL; Beckman Coulter, Brea, CA, USA).

### Statistical analysis

SPSS statistical software for Windows, version 21.0 (SPSS, Chicago, IL, USA) was used for statistical analysis. AEs and baseline hematological indices are presented as categorical variables. The median PFS and OS were determined by the Kaplan-Meier method and were compared among different groups using the log-rank test. We performed Cox proportional hazards regression analyses with stepwise variable selection to identify significant independent prognostic factors for PFS and OS. Hazard ratios (HRs) and 95% confidence intervals (CIs) were generated. All *P* values were 2-sided, and *P* < 0.05 was considered statistically significant.

## Results

### Baseline data and efficacy

**Figure 1:**
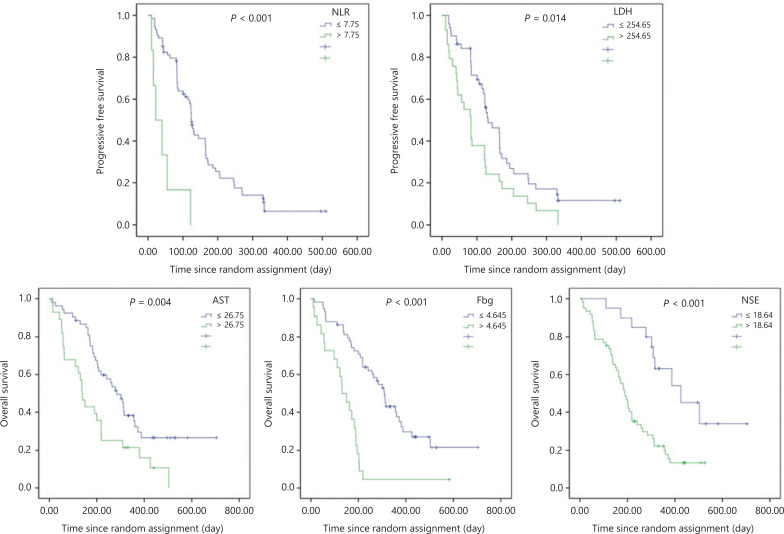
Progression-free survival and overall survival with basal factors in the anlotinib group (multivariate analysis).

First, we found that the cut-off values determined by ROC curves were statistically similar to routine values used for similar time points in clinical practice. The baseline hematological indices obtained from 81 patients in the anlotinib group were analyzed using univariate analysis, which showed that some baseline hematological indices were related to the PFS and OS (**Table 1 and Figure 1**). In the placebo group, analyses of the impact for the PFS and OS were not completed because the median PFS was only 21 days and the median OS was only 146 days. We also detected the distribution of T-lymphocyte subsets in the peripheral blood of patients at our site. Peripheral blood samples were collected from 20 patients (14 in the anlotinib group and 6 in the placebo group). Additionally, we analyzed baseline data (T-lymphocyte subsets) from patients in the anlotinib group. We found that patients with CD8^+^T ≤ 37.5% showed a trend towards a longer PFS compared to others (155 ± 32 days *vs.* 50 ± 18 days, *P* = 0.053), but the difference was statistically insignificant (data not shown).

**Table 1:** Univariate Cox analysis of progression-free survival and overall survival in patients with small-cell lung cancer

Clinical characteristics		Number		PFS (days)	OS (days)	HR (95% CI)		*P* value	
PFS	OS	PFS	OS			PFS	OS	PFS	OS
RBC (×10^12^/L)						–	0.451	–	0.004
–	≤ 3.95 > 3.95	–	45 35	–	215 ± 21 379 ± 41		(0.261, 0.778)		
Hb (g/L)						–	0.465	–	0.023
–	≤ 131 > 131	–	61 19	–	248 ± 22 421 ± 55		(0.240, 0.899)		
Serum albumin (g/L)						–	0.548	–	0.029
–	≤ 38.9 > 38.9	–	24 57	–	193 ± 33 326 ± 30		(0.319, 0.939)		
CEA (μg/L)						–	2.406	–	0.002
–	≤ 11.82 > 11.82	–	59 22	–	339 ± 33 182 ± 20		(1.374, 4.213)		
NSE (μg/L)						–	3.253	–	< 0.001
–	≤ 26.42 > 26.42	–	26 55	–	444 ± 48 210 ± 19		(1.742, 6.077)		
Blood glucose (mmol/L)						1.705	–	0.028	–
≤ 5.395 > 5.395	–	46 35	–	177 ± 22 116 ± 14	–	(1.058, 2.747)			
Plasma globulin (g/L)						1.793	–	0.042	–
≤ 28.36 > 28.36	–	40 28	–	205 ± 33 126 ± 16	–	(1.020, 3.152)			
WBC (×10^9^/L)						1.694	–	0.034	–
≤ 5.135 > 5.135	–	37 43	–	181 ± 22 119 ± 14	–	(1.041, 2.758)			
Neutrophils (×10^9^/L)						1.699	2.023	0.032	0.008
≤ 3.52 > 3.52	38 42	183 ± 22 115 ± 14	326 ± 26 239 ± 32	(1.048, 2.753)	(1.198, 3.415)		
GGT (U/L)						2.255	2.199	0.002	0.004
≤ 39.5 > 39.5	36 >42	201 ± 25 105 ± 14	347 ± 31 230 ± 31	(1.337, 3.804)	(1.287, 3.757)		
INR						2.378	2.344	0.002	0.003
≤ 1.045 > 1.045	60 21	171 ± 18 89 ± 14	332 ± 31 187 ± 32	(1.337, 3.804)	(1.503, 4.273)		
PT (s)						1.966	2.218	0.011	0.008
≤ 11.25 > 11.25	30 51	197 ± 28 121 ± 13	364 ± 41 239 ± 25	(1.171, 3.302)	(1.227, 4.009)		
NLR						5.882	2.198	< 0.001	0.003
≤ 7.75 > 7.75	≤ 4.03 > 4.03	74 6	45 35	160 ± 15 44 ± 17	363 ± 36 209 ± 27	(2.417, 4.314)	(1.310, 3.686)		
AST (U/L)						1.904	2.098	0.021	0.004
≤ 19.15 > 19.15	≤ 26.75 > 26.75	25 56	53 28	211 ± 35 123 ± 12	350 ± 33 193 ± 29	(1.103, 3.287)	(1.252, 3.516)		
LDH (U/L)						1.835	2.440	0.014	0.002
≤ 254.65 > 254.65	≤ 210 > 210	51 29	34 46	181 ± 21 104 ± 17	406 ± 45 213 ± 21	(1.132, 2.973)	(1.404, 4.240)		
Fbg (g/L)						1.817	3.720	0.026	< 0.001
≤ 3.395 > 3.395	≤ 4.645 > 4.645	26 55	59 22	200 ± 29 126 ± 15	348 ± 31 147 ± 24	(1.073, 3.075)	(2.111, 6.557)		

“−” indicates an index that is not a significant factor. PFS, progression-free survival; OS, overall survival; HR, hazard ratio; RBC, red blood cell; Hb, hemoglobin; CEA, carcinoembryonic antigen; NSE, neuron-specific enolase; WBC, white blood cell; GGT, glutamyl transpeptidase; INR, international normalized ratio; PT, prothrombin time; NLR, neutrophil to lymphocyte ratio; AST, aspartate aminotransferase; LDH, lactate dehydrogenase; Fbg, fibrinogen.

### AEs and efficacy in the anlotinib group

**Figure 2:**
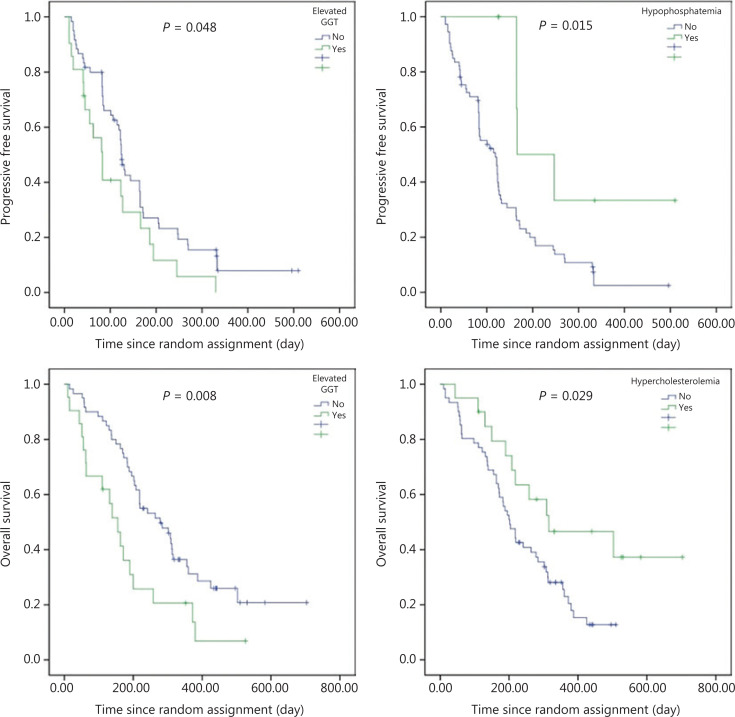
Progression-free survival and overall survival with adverse reactions in the anlotinib group (multivariate analysis).

AEs were defined based on the CTCAE 4.03 (hand-foot syndrome, oral mucositis, headache, hypoproteinemia, rash, etc.). We preliminarily selected 54 AEs that occurred within 2 therapeutic cycles. Patients were categorized into 2 groups: cases with AEs and cases without AEs. Univariate analysis showed that 9 AEs were related to the PFS or OS (**Table 2 and Figure 2**). However, other AEs including elevated thyroid-stimulating hormone (TSH), hypertension, hypertriglyceridemia, and hand-foot syndrome were not significantly correlated with the prognoses. In the placebo group, the above-described indices were not analyzed for impacts on the PFS and OS because patients did not survive for a sufficient duration of time.

**Table 2:** Univariate Cox analysis of PFS and OS with adverse events in the anlotinib group

AEs	Number	PFS (days)	OS (days)	HR		*P* value	
				PFS	OS	PFS	OS
Oral mucositis				0.368	–	0.009	–
Yes No	15 66	261 ± 40 131 ± 14	–	(0.175, 0.776)			
Leukopenia				0.551	–	0.044	–
Yes No	19 62	210 ± 37 128 ± 12	–	(0.308, 0.983)			
Elevated NLR				1.790	–	0.020	–
Yes No	30 51	106 ± 16 175 ± 19	–	(1.096, 2.923)			
Hypophosphatemia				0.283	–	0.015	–
Yes No	8 73	293 ± 63 132 ± 13		(0.102, 0.781)			
Hypercholesterolemia				–	0.476	–	0.029
Yes No	20 61		414 ± 58 237 ± 19	(0.244, 0.928)		
Elevated lipase				–	2.214	–	0.040
Yes No	20 61		172 ± 35 311 ± 28	(1.035, 4.738)		
Hypoproteinemia				–	2.882	–	0.004
Yes No	10 71		133 ± 33 316 ± 27	(1.396, 5.950)		
Elevated amylase				–	3.745	–	0.008
Yes No	5 76		134 ± 19 307 ± 27	(1.415, 9.909)		
Elevated GGT				1.720	2.139	0.048	0.008
Yes No	21 60	107 ± 20 164 ± 18	180 ± 32 331 ± 30	(1.005, 2.943)	(1.224, 3.738)		

“−” means this index is not a significant factor. AEs, adverse events; TSH, Thyroid stimulating hormone.

### Multivariate analysis

For hematological indices at baseline, multivariable analysis indicated that neutrophil to lymphocyte ratio (NLR) > 7.75 (44 ± 17 days *vs.* 160 ± 15 days, HR = 5.882, *P* < 0.001) and lactate dehydrogenase (LDH) > 254.65 U/L (104 ± 17 days *vs.* 181 ± 21 days, HR = 1.835, *P* = 0.014) were independent risk factor of the PFS; aspartate aminotransferase (AST) > 26.75 U/L (193 ± 29 days *vs.* 350 ± 33 days, HR = 2.098, *P* = 0.004), neuron specific enolase (NSE) > 18.64 ng/mL (210 ± 19 days *vs.* 444 ± 48 days, HR = 3.253, *P* < 0.001) and fibrinogen (Fbg) > 4.645 g/L (147 ± 24 days *vs.* 348 ± 31 days, HR = 3.720, *P* < 0.001) were independent risk factors for OS.

For the AEs, multivariate analysis confirmed that elevated GGT was an independent risk factor for the PFS (107 ± 20 days *vs.* 164 ± 18 days, HR = 1.720, *P* = 0.048) and OS (180 ± 32 days *vs.* 331 ± 30 days, HR = 2.139, *P* = 0.008). Hypophosphatemia was a protective factor for the PFS (293 ± 63 days *vs.* 132 ± 13 days, HR = 0.283, *P* = 0.015), and hypercholesterolemia was an independent protective factor (414 ± 58 days *vs.* 237 ± 19 days, HR = 0.476, *P* = 0.029) for the OS (**Table 3**).

**Table 3:** Multivariate Cox regression analysis of PFS and OS in patients with SCLC

PFS	B	SE	Wald	Sig	Exp (B)	95% CI	
NLR (≤ 7.75, > 7.75)	2.306	0.648	12.673	0.000	10.030	2.818	35.691
LDH (≤ 254.65 U/L, > 254.65 U/L)	0.736	0.375	3.847	0.050	2.087	1.001	4.352
Elevated GGT (Yes/no)	0.792	0.304	6.788	0.009	2.207	1.217	4.003
Hypophosphatemia (Yes/no)	–1.223	0.583	4.477	0.034	0.291	0.093	0.913
OS	B	SE	Wald	Sig	Exp(B)	95% CI
AST (≤ 26.75 U/L, > 26.75 U/L)	0.781	0.313	6.217	0.013	2.184	1.182	4.037
NSE (≤ 26.42 ng/mL, > 26.42 ng/mL)	1.065	0.391	7.439	0.006	2.902	1.350	6.240
Fbg (≤ 4.645 g/L, > 4.645 g/L)	0.828	0.423	3.830	0.050	2.288	1.000	5.241
Elevated GGT (Yes, no)	1.344	0.397	11.478	0.001	3.836	1.762	8.348
Hypercholesterolemia (Yes, no)	–0.941	0.361	6.778	0.009	0.390	0.192	0.793

## Discussion

SCLC is the most aggressive malignancy and has a high recurrence due to drug resistance. There has been no therapeutic breakthrough in SCLC for the past 30 years^2^, particularly in second-line or subsequent treatment. The mutation rate of EGFR in SCLC is very low^13^ and, therefore, there is little opportunity for molecular targeted therapy. Tumor angiogenesis inhibitors (e.g., sunitinib, thalidomide, and sorafenib) have also failed in clinical trials^14,15^. For third-line therapy of SCLC patients, the best approach remains to be identified. ALTER 1202 was the first randomized, placebo-controlled trial on refractory SCLC; patients underwent more than 2 protocols of chemotherapy and showed significant improvement in the PFS and, most notably, increased the OS to 218 days. Additionally, the proportion of patients that received further treatments after the end of the trial was smaller in the anlotinib group, which suggested that anlotinib contributed to prolonged survival. However, because anlotinib is a relatively new drug, it remains difficult to predict which patients will benefit most from anlotinib treatment. In the present study, we therefore evaluated the usefulness of baseline hematological indices and AEs for predicting the efficacy and prognosis after treatment with anlotinib.

It has been shown that the NLR before treatment has prognostic significance in NSCLC patients. Previous studies have shown that NSCLC patients with normalized NLR (NLR < 5) after a single chemotherapy cycle have better outcomes^16^. Our previous study indicated that high post-therapeutic peripheral blood NLR was an independent risk factor for PFS in NSCLC patients treated with anlotinib^12^. However, limited data are available for SCLC patients, especially those treated with anti-angiogenic therapy. In the present study, we found that the pre-therapeutic (baseline) high NLR was associated with a shorter PFS and OS. In addition, increased peripheral blood NLR after treatment with anlotinib (AEs) was also a risk factor for PFS. Overall, the OS of patients with an elevated NLR during treatment was poor, but the difference was not statistically significant (238 ± 34 days *vs.* 320 ± 32 days, *P* = 0.193). This finding was likely explained by the fact that NLR was influenced by different follow-up treatments (such as chemotherapy), which weakened its predictive power with respect to the OS.

Previous reports have proposed the concept of “tumor-related leukocytosis”, which is a sign of absent immune function and a lack of lymphocytes^17^. Elevated NLR is associated with a relative deficiency in lymphocytes and insufficient immune function. Hald et al.^18^ found that decreased CD4^+^/CD8^+^ in the tumor tissue matrix was an independent factor for adverse prognoses of NSCLC patients. However, the relationship between lymphocyte subtype and prognoses in SCLC patients has not been clearly defined. We speculated that the efficacy of anlotinib treatment was correlated with immune status, because anlotinib is a robust inhibitor of VEGF, which is an important immunosuppressive factor that plays a vital role in cytotoxic T cells and dendritic cells (DCs)^19^. Several studies have shown that VEGF inhibited the maturation and function of DCs and suppressed the anti-tumor immune response by increasing regulatory T (Treg) cells. Moreover, the proportion of mature DCs can be increased while Treg cells can be reduced with anti-VEGF treatment, which signified an improved tumor immunological microenvironment^20–22^. Our previous study found that anlotinib inhibited tumor growth *via* amelioration of the immune microenvironment^23^. Chu et al.^24^ found that combination treatment with sintilimab and anlotinib showed encouraging efficacy in patients with advanced NSCLC. We have also started a clinical study of immunotherapy combined with anlotinib to determine the synergistic effect of anlotinib with immunotherapy. In this trial, we also found that patients with basal CD8^+^T cells > 37.5% seemed to have a worse PFS. However, CD8^+^T cells contain heterogeneous clusters that include suppressor T (TS) cells and cytotoxic T lymphocytes (TCs). The former are regulatory T cells, the latter are effector T cells. TS cells can inhibit the formation of self-tolerant T cell clones in the thymus, and can also inhibit the immune response during non-antigen penetration. It has been previously shown that TS cells played an important role in abnormal immune function and, so we speculated that TS cells in the CD8^+^T cell population were likely the cause of the poor prognosis. However, we did not further analyze the CD8^+^T cell subsets because data from only 14 cases from our site were obtained. This index was not originally considered as an observable trial metric because, prior to the trial, anlotinib was not known to affect the immune status. Based on these observations, we are analyzing lymphatic subsets in the peripheral blood and infiltration in tumor tissue combined with immunotherapy in a new trial of anlotinib. The study will include analyses of CD45^+^CD4^+^CD25^+^FoxP3^+^T cells and CD25^+^FoxP3^+^T cells in the peripheral blood, as well as the distribution of CD8^+^ and Foxp3^+^ (immunoreactive/suppressor) T cells in different regions of tumor lesions.

One of the characteristics of cancer is metabolic reprogramming. We therefore also focused on the biochemical indices. The baseline GGT level has been shown to have independent prognostic value in various types of cancer^25^. The pro-oxidants derived by GGT can modulate important redox-sensitive processes and functions of the cell, with particular importance on proliferative/apoptotic balance, which has obvious and important implications in tumor progression and drug resistance^26^. This suggests that the GGT level may be related to the prognosis. In the present study, we found that basal GGT > 39.5 U/L was a poor prognostic indicator for both the PFS and OS. In addition, elevated GGT after treatment was an independent risk factor for both the PFS and OS. However, further evidence is required to confirm whether GGT elevation is a cause of anlotinib resistance. We also found that a higher LDH level at baseline was an independent risk factor for the PFS, but not for the OS. Diem et al.^27^ reported that in 66 melanoma cases treated with PD-1 inhibitors, a greater than 10% increase in LDH was significantly associated with a shorter OS, indicating the potential value of LDH as a prognostic marker. However, in our study, no significant correlation was found between changes in LDH during treatment or the prognosis. This may be explained by the fact that we divided patients into groups with and without LDH elevation, but did not analyze according to the degree of LDH increase. We found that high pre-therapeutic serum AST level was associated with a poor clinical outcome, and Cox regression analysis showed that AST was an independent prognostic factor for a poor OS. Zhang et al.^28^ reported AST/ALT as independent factors for predicting the OS of primary hepatic cancer patients. However, the relationship of AST/ALT with prognosis in lung cancer has not been well studied. Further studies are therefore required to determine the ability AST/ALT to predict a curative effect and overall prognosis with anlotinib treatment. In addition, hypophosphatemia caused by anlotinib was an independent prognostic factor for PFS, and subsequent hypercholesterolemia was an independent prognostic factor for OS. Our findings implied that anlotinib may inhibit tumor cell proliferation through alterations in cell metabolism. This resulted in altered lipid metabolism blood indices, despite the changes in the above metabolic indices, which may also be caused by hepatic metabolism because slight changes in hepatic function were identified during treatment. Considering the broad spectrum function of anlotinib, it is important to determine whether it is a metabolism-related drug and if any of the aforementioned indices can serve to predict its efficacy. Further studies should be conducted to determine the effect of anlotinib on hepatic metabolism, and to determine if there is a need for clinical trials of metabolic drugs in combination with anlotinib.

Our analysis showed that elevated prothrombin time (PT), international normalized ratio (INR), and Fbg at baseline were associated with decreased survival; Fbg was an independent prognostic factor for the OS. Fan et al.^29^ showed that elevated plasma Fbg was an independent factor associated with poor outcomes in SCLC patients; other studies have reported that prolongations of PT and INR were associated with a poor prognosis^30^. The blood coagulation index may therefore be useful in predicting clinical outcome, survival, and treatment response in patients with lung cancer. However, because anlotinib is a vascular targeting drug that affects coagulation, the ability of the aforementioned markers to predict anlotinib efficacy remains to be determined, and further studies are required to eliminate possible confounding factors.

In the present study, the incidence of hypertension was 40.7% (33/81), hand-foot syndrome was 25.9% (21/81), and oral mucositis was 18.51% (15/81), while skin rash occurred at low frequencies. Oral mucositis (*P* = 0.009) and hypertension (*P* = 0.109) during the treatment were associated with a longer PFS. There was no significant correlation between the occurrence of hand-foot syndrome and survival. These results are in disagreement with other study results^16^ and were likely due to a smaller pool of cases in this study, when compared to others.

In conclusion, the efficacy and prognosis of anlotinib treatment in SCLC patients may be predicted by several factors, including baseline and treatment values of laboratory-measured indices. These predictive indicators may be used to screen for optimal therapeutic patient populations to achieve maximal benefits with anlotinib treatment. Further studies should be conducted to identify the most reliable markers for efficacy and prognosis, and to elucidate the basic mechanism of anlotinib action in SCLC patients.

## Limitations

All included patient data were from clinical studies and, therefore, it was impossible to detect and analyze the corresponding indicators in local tumor lesions. In addition, due to the small number of cases, we could not further analyze subsets of CD8^+^T cells. Although our previous basic studies reported that anlotinib improved the immune microenvironment to inhibit tumor growth, further research is needed to confirm these findings. Moreover, the potential mechanism of anlotinib action, and specifically its effect on lipid metabolism, should be further characterized.
